# Assessment of specific risk scores for patients admitted to the ICU for severe community-acquired pneumonia

**DOI:** 10.1186/cc14089

**Published:** 2015-03-16

**Authors:** C Joya-Montosa, MD Delgado-Amaya, E Trujillo-García, E Curiel-Balsera

**Affiliations:** 1 Hospital Regional de Málaga, Spain

## Introduction

The aim of the study is to evaluate the calibration and discrimination of two specific risk scores for community-acquired pneumonia (CAP) in patients with this illness who required ICU admission.

## Methods

A retrospective descriptive study of patients with severe CAP admitted to the ICU between January 2008 and September 2013. We analyzed clinical and epidemiological variables and APACHE II, SAPS III, CURB-65 and Pneumonia Severity Index (PSI) that were recorded in the first 24 hours. We used the Student *t *test to compare means and the chi-square test for univariate analysis. The standardized mortality ratio (SMR) and Hosmer-Lemershow test were calculated to analyze the calibration and ROC curve analysis for discrimination of different scores.

## Results

**Figure 1:**
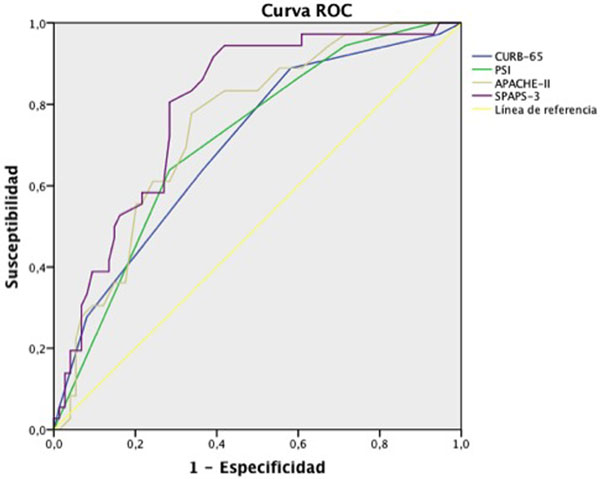


We analyzed 111 patients aged 57.5 ± 17.7 years, with 63.1% (70) males. The APACHE II score at admission was 19.8 ± 17.7 and SAPS III was 60.6 ± 16.7. ICU mortality was 29.7% (33). There was association between the four scores and mortality. The SMR for APACHE II was 0.87 and 0.85 for the SAPS III. Figure 1 shows the ROC curve for the four scores, the best observed discrimination obtained was for SAPS III score (AuC 0.79) and the worst was obtained for CURB-65 score (AuC 0.7). The Hosmer-Lemeshow test showed acceptable calibration for the four predictive systems (*P >*0.05).

## Conclusion

The four analyzed scores presented good calibration, but discrimination seems better in SAPS III. Given the difficulty of calculating PSI, and its low discrimination (similar to CURB-65), we prefer to use the CURB-65 score in routine clinical practice.

